# Polysaccharides From the Aerial Parts of *Tetrastigma Hemsleyanum* Diels et Gilg Induce Bidirectional Immunity and Ameliorate LPS-Induced Acute Respiratory Distress Syndrome in Mice

**DOI:** 10.3389/fphar.2022.838873

**Published:** 2022-03-11

**Authors:** Jingjing Lu, Bingqi Zhu, Fangmei Zhou, Xinghong Ding, Chaodong Qian, Zhishan Ding, Xiaoqing Ye

**Affiliations:** ^1^ College of Life Science, Zhejiang Chinese Medical University, Hangzhou, China; ^2^ School of Medical Technology and Information Engineering, Zhejiang Chinese Medical University, Hangzhou, China; ^3^ School of Basic Medical Sciences, Zhejiang Chinese Medical University, Hangzhou, China

**Keywords:** polysaccharide, TLR2, TLR4, bidirectional immunity, ARDS, Tetrastigma hemsleyanum diels et gilg

## Abstract

*Tetrastigma hemsleyanum* Diels et Gilg (Sanyeqing, SYQ) has traditionally been used to treat inflammation, high fever and improve immune function of patients. Polysaccharides have been proved to be one of the important components of SYQ. Previous studies have confirmed the antipyretic and antitumor effects of polysaccharides from SYQ (SYQP), and clarified that SYQP could enhance immunity through TLR4 signalling pathway. However, there were more possibilities for the mechanism by which SYQP exerted immunomodulatory effects and the role of SYQP in acute respiratory distress syndrome (ARDS) is elusive. The purpose of this study was further to explain the bidirectional modulation of immunity mechanism of SYQP *in vitro* and its effect in LPS-induced ARDS *in vivo*. Experimental results showed that SYQP significantly stimulated gene expressions of TLR1, TLR2 and TLR6 and secretion of cytokines in RAW264.7 cells. Individual or combined application of TLR2 antagonist C29 and TLR4 antagonist TAK-242 could reduce SYQP-mediated stimulation of cytokine secretion in RAW264.7 cells and mouse peritoneal macrophages (MPMs) to varying degrees. On the other hand, SYQP markedly inhibited the expression levels of inflammatory cytokines, NO, iNOS and COX-2 in LPS-treatment RAW264.7 cells. Moreover, *in vivo* results indicated that SYQP significantly reduced LPS-induced damage in ARDS mice through alleviating LPS-induced pulmonary morphological damage, inhibiting myeloperoxidase (MPO) expression levels, ameliorating the inflammatory cells in bronchoalveolar lavage fluid (BALF) and improving hematological status. Meanwhile, SYQP evidently reduced IL-6, TNF-α and IFN-γ secretion, the overexpression levels of TLR2 and TLR4, as well as the phosphorylation of NF-κB p65. In addition, SYQP reduced the phosphorylation of JAK2 and STAT1 and the overexpression of NLRP3, caspase-1, caspase-3 and caspase-8 in lung tissues of ARDS mice. In summary, our study confirmed that SYQP induced bidirectional immunity and ameliorated LPS-induced acute respiratory distress syndrome in mice through TLR2/TLR4-NF-κB, NLRP3/caspase and JAK/STAT signaling pathways, which provided a theoretical basis for further use of SYQP.

## Introduction

To date, the coronavirus disease 2019 (COVID-19) pandemic has resulted in over 4.8 million deaths. Cytokine storms caused by expressive inflammatory factors, acute lung injury and severe acute respiratory distress syndrome (ARDS) were the main characteristics of COVID-19 (Jafarzadeh et al., 2020). Among them, ARDS could cause multiple organ failure, and was the major cause of mortality in COVID-19 patients (Pooladanda et al., 2019). Therefore, inhibition of ARDS was of great significance in alleviating body injury, which might also be helpful for the treatment of COVID-19. According to the current clinical guidelines in China and experiences of treating patients with severe epidemic diseases, such as severe acute respiratory syndrome (SARS) (Liu et al., 2004; Li and Peng, 2013; Li and De Clercq, 2020), traditional Chinese medicine (TCM) is used for the prevention and therapy of ARDS of COVID-19 patients in China. In this regard, the pharmacodynamics and mechanisms of TCM have attracted widespread research attention.

*Tetrastigma hemsleyanum* Diels et Gilg, which is known as Sanyeqing (SYQ) in China, belongs to the grape family Vitaceae and is a valuable Chinese medicinal herb. The aerial parts, leaves and root tubers of SYQ are clinically used to treat inflammation and immune-related diseases (Xiong et al., 2015; Russell et al., 2020; Ruyi Zhu et al., 2020). It has been reported that Chinese herbal preparations containing SYQ can be used for the clinical treatment of COVID-19, for example, Jinlian disinfection drink and Hua Shi Xuan Fei mixture (Ji et al., 2021). Due to its good pharmacological activity, SYQ has been designated as one of the new “eight well-known TCMs in Zhejiang Province”.

In folk medicine, decoction with water is the traditional preparation method of TCMs. As one of the important water-soluble components, polysaccharides have attracted the attentions of the researches for its pharmacological functions and mechanisms in treating inflammation and immune-related diseased (Wang et al., 2014; Sun et al., 2017; Zhang et al., 2018; Wang et al., 2020). Preliminary studies have shown that some plant polysaccharides act as natural modulators and stimulate the immune response by activating TLR receptors (Zhou et al., 2017), regulating the production of antibodies, and promoting the release of cytokines, such as IL-6, IFN-γ, TNF-α and nitric oxide (NO) (Zheng et al., 2017), without causing significant side effects (Schepetkin and Quinn, 2006). Our previous study revealed that polysaccharides from the aerial part of *T. hemsleyanum* (SYQP) have antipyretic and antitumour effects in mice (Bingqi Zhu et al., 2020), on the other hand, SYQP could enhance immune responses by activating the TLR4 signaling pathway at the receptor level (Zhou et al., 2021). However, it remained unclear regarding to the roles of other TLR receptors when interacting with SYQP and the effects of SYQP treatment on the mice with LPS-induced ARDS have rarely been investigated. Based on previous studies, we hypothesized that SYQP might have dual immunomodulatory effects through different TLRs and treat LPS - induced ARDS in mice. The mechanism underlying the protective immunity of SYQP is worthy of further study.

This study aimed to investigate the protective immunity and molecular mechanism of SYQP *in vivo* and *in vitro*. To achieve the goal, we analysed the mechanism of the TLR-mediated modulation of SYQP-induced macrophage responses using the RAW264.7 cells and mouse peritoneal macrophages (MPMs). In addition, we used LPS to induce inflammation in RAW264.7 cells and ARDS in mice to investigate the anti-inflammatory effects and possible mechanism of SYQP.

## Materials and Methods

### Materials and Regents

The aerial parts of *Tetrastigma hemsleyanum* Diels et Gilg were obtained from Hangzhou China Agrotime Agri-Tech Co., Ltd. SYQP was prepared and characterized in our laboratory as previously reported (Bingqi Zhu et al., 2020).

3-(4,5-dimethylthiazol-2-yl)-2,5-diphenyltetrazolium bromide (MTT) (#M2128-100 MG), dimethyl sulfoxide (DMSO) (#V900090), LPS (#l2880-100 mg), dexamethasone (DEX) (#D4902-100 MG) and Pam3CSK4 (P3C) (#tlrl-pms) were purchased from Sigma Chemical Co. (MO, United States). TAK-242 (#HY-11109-10 mg) and C29 (C_16_H_15_NO_4_) (#HY-100461-5 mg) were purchased from MedChemExpress (MCE) (NJ, United States). Peroxidase-conjugated goat anti-mouse IgG (#115–035–003) and goat anti-rabbit IgG (#111–035–003) were purchased from Jackson ImmunoResearch (PA, United States). RNA-Quick Purification Kit (#RN001) was purchased from ESscience Biotech (Shanghai, China). A BeyoRT™ II First Strand cDNA Synthesis Kit (#D7170M) was purchased from Beyotime Biotechnology (Shanghai, China). PowerUp™ SYBR™ Green Master Mix (#A25742) was purchased from Thermo Fisher Scientific (MA, United States), ROS Assay Kit (2′,7′-dichlorofluorescin diacetate (DCFH-DA) (#S0033S) was purchased from Beyotime Biotechnology (Shanghai, China).

### Cell Culture

RAW264.7 murine macrophages were purchased from Shanghai Chinese Academy of Sciences cell bank (Shanghai, China). Purified MPMs from Balb/c mice were aseptically harvested based on a reference protocol (Liu et al., 2006). The RAW264.7 cells and extracted MPMs were cultured in DMEM with a high sugar content supplemented with 10% FBS and maintained at 37°C in a humidified 5% CO_2_ atmosphere.

### Cell Viability Analysis

MTT assay was used to evaluated cell viability. Briefly, RAW264.7 cells were collected and seeded in 96-well plates (1 × 10^4^ cells/well) for 12 h and then treated with SYQP (0, 0.1, 1,10,100,1000 μg/ml) for 24 h. After incubation, 20 μL MTT solution was added to each well to a final concentration of 0.5 mg/ml. After further incubation for 4h, the supernatant was removed and 150 μL DMSO was subsequently added to each well. The absorbance at 570 nm was measured with a microplate reader.

### Reactive Oxygen Species Analysis

ROS levels in RAW264.7 cells was detected with ROS assay kit. Briefly, RAW264.7 cells were pre-treated with SYQP (25, 50, 100 μg/ml) or DEX (4 μg/ml) for 2 h, and followed by stimulation with or without LPS (1 μg/ml) for 18 h. After treatment, DCFH-DA was added to the cultured cells for 20 min, and the nucleus were stained with 4′,6-diamidino-2-phenylindole (DAPI) for 10 min in the dark. After washing three times with PBS, cells were photographed by Nikon ECLIPSE Ti-DH inverted fluorescence microscope (Tokyo, Japan).

### Animal Administration

Healthy male Balb/c mice weighing 20–25 g were purchased from the Laboratory Animal Centre of Zhejiang Chinese Medical University (Hangzhou, China). The ethical approval number of the animal model study is IACUC-20210802–13. The mice were raised under standard conditions. All experimental procedures were in accordance with the People’s Republic of China (PRC) guidelines for the Care and Use of Laboratory Animals and were carried out strictly in accordance with the Guidelines of Zhejiang Chinese Medical University for Animal Experiments.

### LPS-Induced Acute Respiratory Distress Syndrome in Mice

Animals were divided into six groups: the control group, which was intragastrically administered with normal saline; the LPS group, which was injected intraperitoneally with LPS (20 mg/kg); DEX and three SYQP + LPS groups, which were intragastrically administered with DEX (5 mg/kg), 50 mg/kg SYQP (SYQP of low dose, SYQPL), 75 mg/kg SYQP (SYQP of middle dose, SYQPM) and 150 mg/kg SYQP (SYQP of high dose, SYQPH) for 14 days. LPS was injected intraperitoneally 30 min after the last intragastric administration. Mice were sacrificed 12 h after LPS treatment.

### Bronchoalveolar Lavage Analysis

Mice were fixed, and the skin and muscles in front of the neck were cut to expose the trachea. After endotracheal intubation, BALF were collected with 1 ml ice-cold PBS three times. Then BALF were combined and centrifuged at 3,500 rpm for 15 min. The cell pellets obtained after centrifugation were resuspended in 200 μL PBS and subjected to differential cell counter ADVIA 2120i Hematology System (Siemens, Germany).

### Blood Analysis

Blood was used to determine different blood parameters. The blood was collected in EDTA-K_2_ contained centrifugal tubes. The whole blood was subjected to automatic blood cell analyzer for detailed hematological analysis.

### Measurement of GSH, SOD and MDA Levels

Plasma was collected by centrifugation (12,000 rpm, 10 min, 4°C) of the whole blood and then stored at −80°C until GSH, SOD and MDA measurement.

### Flow Cytometry Assay

The cytokine contents and NO production in the culture supernatants of RAW264.7 cells and plasma of ARDS mice were assayed using a Cytometric Bead Array Mouse Th1/Th2/Th17 Cytokine Kit (BD Biosciences, San Diego, United States) and ELISA kit according to the manufacturer’s instruction, respectively. RAW264.7 cells were processed as follows: cells (2 × 10^5^ cells/well) were seeded in 24-well plates, pre-treated with a series of concentrations of SYQP or DEX (4 μg/ml) for 2 h, and followed by stimulation with or without LPS (1 μg/ml) for 18 h. Alternatively, RAW264.7 cells or MPMs were pre-treated with C29 (25 μM) or TAK242 (10 μM) for 1 h and then cultured with SYQP (100 μg/ml) at 37°C for 18 h. Subsequently, the supernatant was collected by centrifugation (3,000×*g*, 10 min, 4°C) to determine cytokine and NO production.

### Histological Evaluation, Immunohistochemistry and Immunofluorescence

Samples of the middle lobe of right lung were fixed in 4% paraformaldehyde for more than 48 h, dehydrated with graded alcohol, and then embedded in paraffin. Paraffin sections stained with hematoxylin and eosin were used for gross morphology and lung damage and inflammation. Images were captured under a microscope (Eclipse TS100; Nikon Corporation, Tokyo, Japan). A quantitative scoring system, which included alveolar congestion, alveolar hemorrhage, neutrophil infiltration, aggregation in the airspace or vessel wall, alveolar wall/hyaline membrane thickness, and inflammatory cell infiltration, was introduced to evaluate lung injury. The grading scale for the lung tissue pathologic findings was as follows: 0 (normal = no inflammation, no airway thickening, no edema), 1 (minimal cellular infiltration, and minimal edema), 2 (mild-moderate cellular infiltration, plus mild airway thickening and mild edema), 3 (severe cellular infiltration, plus diffuse airway thickening and severe edema) in 10–12 fields per mouse (Xian et al., 2021).

Immunohistochemistry was performed as per standard protocol reported earlier (Xian et al., 2021). Lung sections were subjected to incubation with antibodies specific for myeloperoxidase (MPO). Images were captured under a microscope (Eclipse TS100; Nikon Corporation, Tokyo, Japan). Stained areas were quantified using ImageJ software.

The immunofluorescence assay of RAW264.7 cells and lung sections were performed according to previous study (Tian et al., 2021). Briefly, RAW264.7 cells (5 × 10^5^ cells/well) were seeded in Laser confocal Petri dishes, pre-treated with SYQP (25, 50, 100 μg/ml) or DEX (4 μg/ml) for 2 h, and followed by stimulation with or without LPS (1 μg/ml) for 18 h. After treatment, the nuclear translocation of p65 of RAW264.7 cells was subjected to incubation with antibodies specific for NFκB-p65. Lung sections were subjected to incubation with antibodies specific for F4/80. Nuclei were co-stained for 10 min with 0.1 g/ml DAPI. Images were captured under laser scanning confocal microscope (Zeiss, Oberkochen, Germany) and Nikon ECLIPSE Ti-DH inverted fuorescence microscope (Nikon, Tokyo, Japan), respectively. Stained areas were quantified using ImageJ software.

### Quantitative Reverse Transcription-Polymerase Chain Reaction

Total RNA was isolated from RAW264.7 cells and lung tissues by RNA-Quick Purification Kit. After determining the concentration of the isolated RNA and reverse transcription, PCR was performed in an ABI-7500 Real-Time PCR System. The mRNA expression of each target gene was normalized to GAPDH. The relative fold change was calculated using the 2^-∆∆Ct^ method. All primer sequences (Sangon Biotech, Shanghai, China) are listed in Table 1.

**TABLE 1:** Primers for qRT-PCR.

Gene		Sequences
TLR1	Forward primer	GGA​CCT​ACC​CTT​GCA​AAC​AA
	Reverse primer	GGT​GGC​ACA​AGA​TCA​CCT​TT
TLR2	Forward primer	CTC​CCA​CTT​CAG​GCT​CTT​TG
	Reverse primer	AGG​AAC​TGG​GTG​GAG​AAC​CT
TLR3	Forward primer	AGC​TTT​GCT​GGG​AAC​TTT​CA
	Reverse primer	GAA​AGA​TCG​AGC​TGG​GTG​AG
TLR4	Forward primer	ATG​GCA​TGG​CTT​ACA​CCA​CC
	Reverse primer	GAG​GCC​AAT​TTT​GTC​TCC​ACA
TLR5	Forward primer	CAG​ATT​CCC​TGG​ATC​CTC​AA
	Reverse primer	ACA​GCC​GAA​GTT​CCA​AGA​GA
TLR6	Forward primer	AGT​TGC​CTT​CTT​GGG​ACT​GA
	Reverse primer	TTC​TGC​AAG​TGC​ATC​ATC​GT
TLR7	Forward primer	CCA​CAG​GCT​CAC​CCA​TAC​TT
	Reverse primer	CAA​GGC​ATG​TCC​TAG​GTG​GT
TLR8	Forward primer	GGC​ACA​ACT​CCC​TTG​TGA​TT
	Reverse primer	CAT​TTG​GGT​GCT​GTT​GTT​TG
TLR9	Forward primer	TGC​AGG​AGC​TGA​ACA​TGA​AC
	Reverse primer	TAG​AAG​CAG​GGG​TGC​TCA​GT
IL-6	Forward primer	AGT​TGC​CTT​CTT​GGG​ACT​GA
	Reverse primer	TTC​TGC​AAG​TGC​ATC​ATC​GT
IL-1β	Forward primer	CAA ATC TCG CAG CAG CAC ATC
	Reverse primer	TCA TCT CGG AGC CTG TAG TGC
TNF-α	Forward primer	CGA​GTG​ACA​AGC​CTG​TAG​CCC
	Reverse primer	GGG​CAG​CCT​TGT​CCC​TTG​A
iNOS	Forward primer	CAT​GCT​ACT​GGA​GGT​GGG​TG
	Reverse primer	CAT​TGA​TCT​CCG​TGA​CAG​CC
COX2	Forward primer	TGC​TGT​ACA​AGC​AGT​GGC​AA
	Reverse primer	GCA​GCC​ATT​TCC​TTC​TCT​CC
GAPDH	Forward primer	CAT​CAC​TGC​CAC​CCA​GAA​GAC​T
	Reverse primer	GAC​ACA​TTG​GGG​GTA​GGA​ACA​C

### Western Blot Analysis

Western blot assays were used to investigate the immunomodulatory molecular mechanism of SYQP in LPS-induced RAW264.7 cells and lung tissues of ARDS mice. After incubation, the total proteins were extracted according to the method of literature (Ren et al., 2019). Protein of cell and tissue lysates were quantified by the BCA reagent. Harvested proteins were denatured at 95°C for 10 min, and 50 μg of protein from each sample was electrophoresed by sodium dodecyl sulfate polyacrylamide gel electrophoresis (SDS-PAGE) before transferring onto polyvinylidene difluoride membranes. The membranes were blocked for 1 h at room temperature in 5% non-fat dry milk, and followed by incubation with primary antibodies of iNOS (Cell Signaling Technology [CST], #13120, 1:1,000), COX-2 (Affinity Biosciences, #AF7003, 1:1,000), TLR4 (Sigma-Aldrich, #SAB1300056-100UG, 1:1,000), TLR2 (Sigma-Aldrich, #SAB1300199-100UG, 1:1,000), phospho-NF-κB p65 (CST, #3031L, 1:1,000), NF-κB p65 (Sigma-Aldrich, #ABE347, 1:1,000), *p*-JAK2 (CST, #3771S, 1:1,000), JAK-2 (Bioss, bs-23003R, 1:1,000), p-STAT1 (Immunoway, #YP0249, 1:1,000), STAT1 (Immunoway, #YT4439, 1:1,000), NLRP3 (Affinity Biosciences, #DF7438, 1:1,000), caspase1 (AdipoGen, AG-20B-0042, 1:1,000), caspase3 (CST, #9662, 1:1,000), caspase8 (AdipoGen, AG-20T-0138-C100, 1:1,000), and β-actin (Proteintech, 66009-1-IG, 1:1,000) at 4°C overnight. Horseradish peroxidase (HRP)-conjugated secondary antibody (1:5,000) was incubated for 1 h at room temperature. Finally, the signal was visualized with the ECL Detection Kit, and ImageJ software was used to analysis proteins.

### Statistical Analysis

Data are expressed as the means ± SDs, and differences between groups were examined using ANOVA and Tukey post hoc tests. *p* values less than 0.05 were considered statistically significant.

## Results

### SYQP Regulated TLRs mRNA Expression in RAW264.7 Cells

**FIGURE 1:**
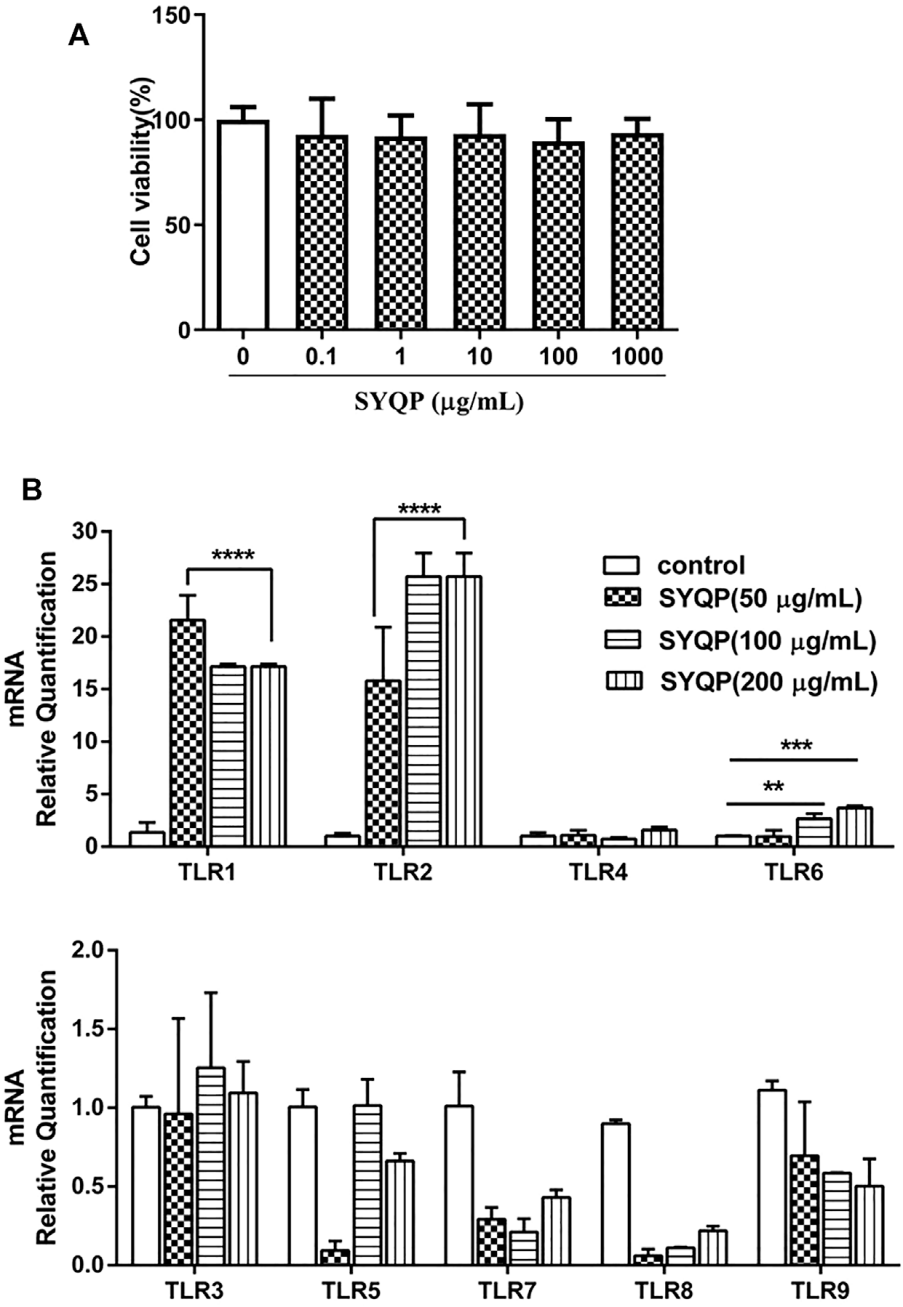
Evaluation of the immune activities of SYQP on RAW264.7 cells. **(A)** RAW264.7 cells were treated with different concentrations of SYQP for 24 h and then detected by MTT assay. **(B)** The TLR gene expression levels in RAW264.7 cells were detected using qRT PCR after 24 h stimulation with SYQP. Data were presented as the mean ± SD (*n* = 9); ****p < 0.0001, ***p < 0.001 and **p < 0.01 versus the control group.

RAW264.7 cell line was derived from Abelson murine leukemia virus-induced tumors in Balb/c mice. MTT assay was used to evaluate the viability of RAW264.7 cells exposed to different concentrations of SYQP. The result showed that SYQP had no significant effect on the viability of RAW264.7 cells within the concentration range of 0–1,000 μg/ml (Figure 1A).

Different TLRs are the commonly studied innate immune system receptors (Root-Bernstein, 2021). To investigate the effect of SYQP on different TLRs, we measured the mRNA expression of TLR1-TLR9 in RAW264.7 cells by qRT-PCR in this study. As shown in Figure 1B, compared with control group, the mRNA expression levels of the TLR1, TLR2 and TLR6 genes were upregulated in SYQP-treated RAW264.7 cells. But there was no significant difference in TLR4 gene expression level compared with the control group, which was consistent with our previous experiment results (Zhou et al., 2021). In addition, the mRNA expression levels of the TLR3, TLR5, TLR7 and TLR9 genes remained unchanged. Similar results have been found in other studies of polysaccharides, for example, cordyceps sinensis polysaccharides were shown to regulate immune activity by upregulating the expression levels of TLR2, TLR4 and TLR6 in mice (Ying et al., 2020), and Salvia miltiorrhiza polysaccharides activated the mRNA expression of the TLR1, TLR2 and TLR4 genes in T lymphocytes (Chen et al., 2017). These data preliminarily indicated that SYQP could not only stimulate TLR4, but also activate immunity by increasing the gene expression of TLR1, TLR2 and TLR6 on the surface of macrophages.

### The TLR2 Antagonist C29 and TLR4 Antagonist TAK-242 Abolished SYQP-Mediated Stimulation of Cytokine Secretion in Macrophages

**FIGURE 2:**
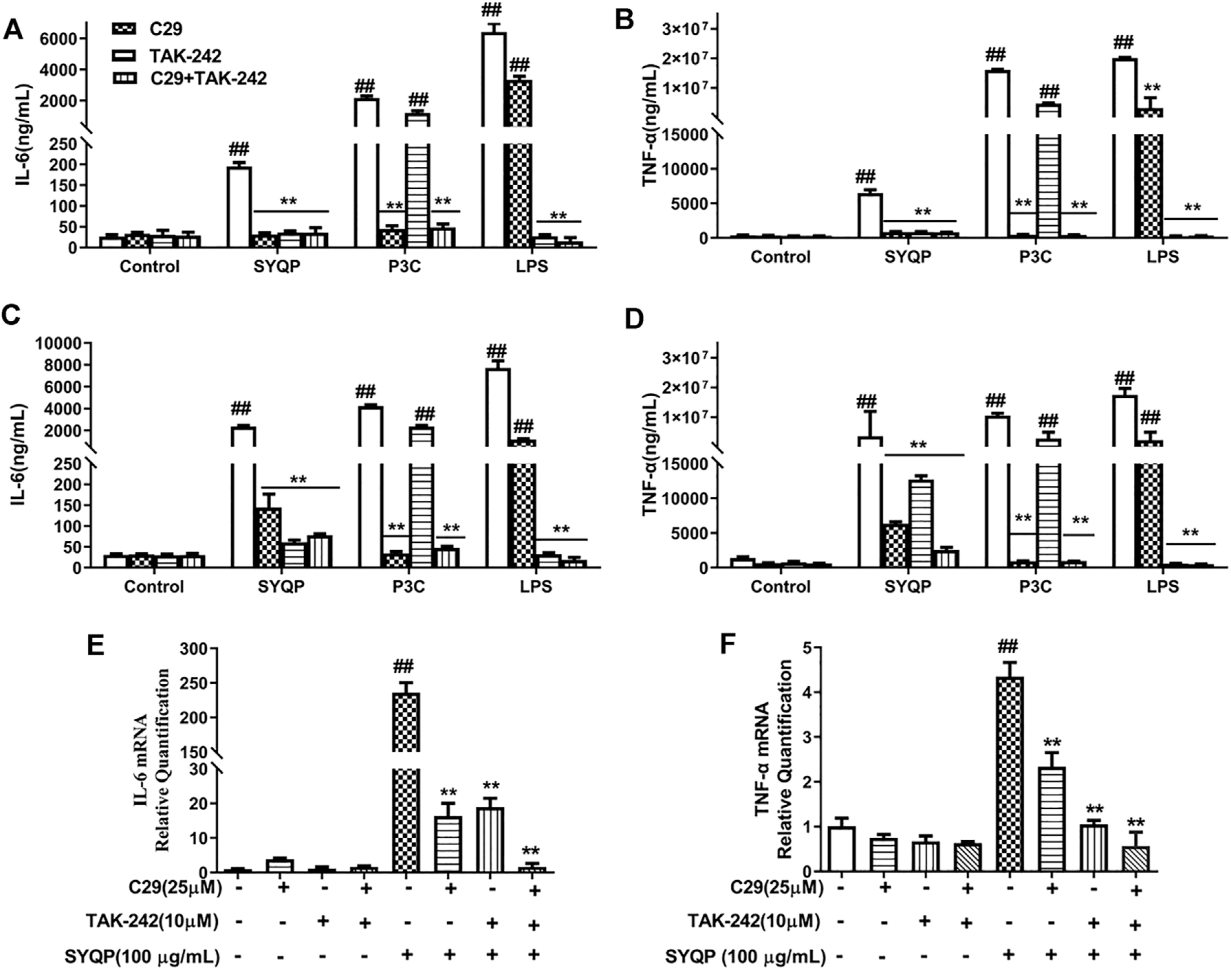
Pro-inflammatory cytokine release and expression in TLR2/TLR4 antagonist-treated RAW264.7 and MPM cells. **(A,B)** RAW264.7 cells were pre-treated with C29 (25 μM) or TAK242 (10 μM) for 1 h and then cultured with SYQP (100 μg/ml) at 37°C for 18 h. Subsequently, the supernatant was collected by centrifugation to determine IL-6and TNF-α production. **(C,D)** MPMs were pre-treated with C29 (25 μM) or TAK242 (10 μM) for 1 h and then cultured with SYQP (100 μg/ml) at 37°C for 18 h. Subsequently, the supernatant was collected by centrifugation to determine IL-6 and TNF-α production. **(E,F)** qRT PCR assay was used to detect the gene expression levels of IL-6 and TNF-α in TLR2/TLR4 antagonist-treated RAW264.7 cells after SYQP stimulation. Data are presented as the mean ± SD (*n* = 6); ^##^ indicates a very significant difference from the control group, *p* < 0.01. ^**^ indicates a very significant difference from the SYQP group, *p* < 0.01.

TLR2 coactivates TLR1 or TLR6, forming heterodimers to evoke effects (Takada and Uehara, 2006). Thus, in this part, we mainly focused on correlation between TLR2/TLR4 and macrophage activation induced by SYQP on TLR2/TLR4 antagonist-treated cells (RAW264.7 and MPMs). TAK-242 and C29, antagonists of TLR4 and TLR2 respectively, have been proven to exhibit effective specific blocking properties (Chen et al., 2018). Firstly, we measured pro-inflammatory cytokines in TLR2/TLR4 antagonist-treated RAW264.7 cells (Figures 2A,B) and MPMs after SYQP stimulation (Figures 2C,D). The known TLR2 and TLR4 ligands Pam3Cys and LPS, respectively, were used as positive controls. In RAW264.7 cells and MPMs, SYQP stimulated the production of IL-6 and TNF-α. Individual application of C29 or TAK-242 decreased the stimulation of IL-6 and TNF-α production caused by SYQP, but cytokine secretion remained at a higher level compared with the control group. Combined application of C29 and TAK-242 almost completely abolished the stimulation of IL-6 and TNF-α production caused by SYQP. In addition, similar to SYQP, LPS induced overexpression of IL-6 and TNF-α in untreated and TLR2 antagonist-treated cells but not TLR4 antagonist-treated cells. In contrast, as expected, P3C was able to induce cytokine overexpression in untreated and TLR4 antagonist-treated cells but not TLR2 antagonist-treated cells.

We further investigated whether C29 or TAK-242 inhibited the transcription of inflammatory cytokines in SYQP-stimulated RAW264.7 cells. As shown in Figures 2E,F, 100 μg/ml SYQP increased the mRNA expression levels of IL-6 and TNF-α in RAW264.7 cells. Compared with SYQP treatment alone, individual treatment and combined treatment of C29 and TAK-242 inhibited the SYQP-induced increase in inflammatory cytokine transcription in RAW264.7 cells. The above experimental results showed that the immunity of SYQP can activate the TLR2 and TLR4 at the receptor level and reveals TLR2/TLR4 modulatory characteristics.

### SYQP Reduced the Release of Inflammatory Cytokines and Oxidative Stress in LPS-Induced RAW264.7 Cells

**FIGURE 3:**
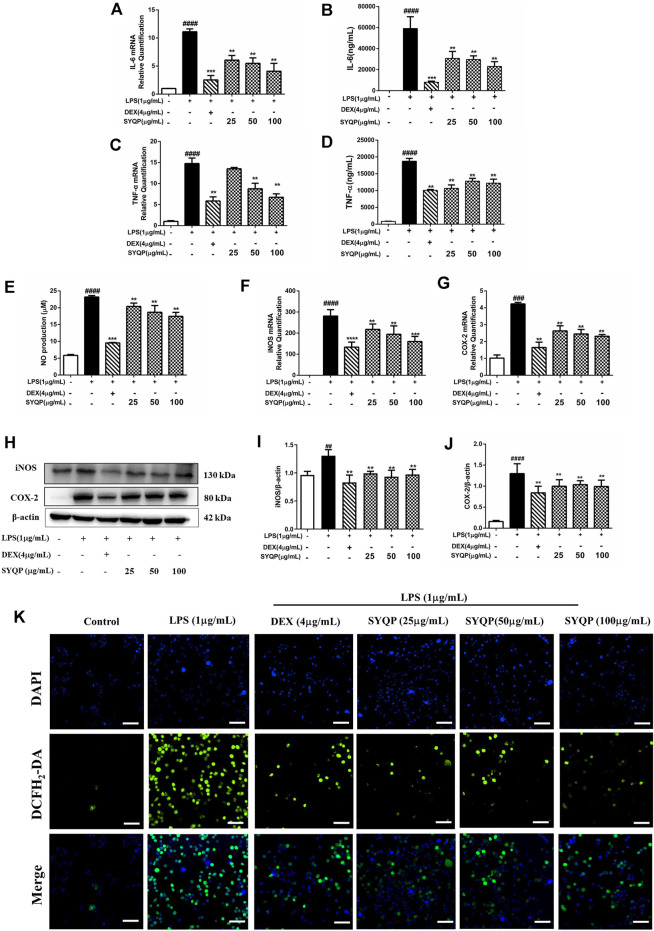
Effects of SYQP on the release of inflammatory cytokines and Oxidative stress in LPS-induced RAW264.7 cells. **(A–D)** RAW264.7 cells were seeded and pre-treated with a series of concentrations of SYQP or DEX (4 μg/ml) for 2 h, and followed by stimulation with LPS (1 μg/ml) for 18 h. Subsequently, flow cytometry and qRT PCR were used to detect the expression levels of IL-6 and TNF-α, respectively. **(E)** Effects of SYQP on the production of NO in LPS-treated RAW264.7 cells were measured by ELISA assay. **(F–J)** RAW264.7 cells were treated in previous mentioned ways, and the gene and protein expression of iNOS and COX-2 were examined. **(K)** Immunofluorescence staining was used to detect the effects of SYQP on reactive oxidative stress level in LPS-induced RAW264.7 cells. Green fluorescence represents DCFH2-DA-labeled reactive oxidative stress and blue fluorescence represents DAPI-labeled nucleus. The bars represent the mean ± SD (*n* = 3); ####*p* < 0.0001, ###*p* < 0.001 and ##*p* < 0.01 versus the control group; ***p < 0.001 and **p < 0.01 versus the model group.

In this part, we further performed multiplex analysis *in vitro* and *in vivo* to determine the anti-inflammatory effects and the underlying mechanism. Initially, we examined the effect of SYQP on LPS-induced oxidative stress and inflammatory damage *in vitro*. RAW264.7 cells were pre-treated with SYQP or DEX for 2 h, and followed by stimulation with LPS for 18 h. The results demonstrated that SYQP played anti-inflammatory roles by suppressing the transcriptions and releases of IL-6 and TNF-α (Figures 3A–D). As shown in Figure 3E, SYQP effectively reduced NO secretion in LPS-induced RAW264.7 cells, and the mRNA and protein results showed that pre-treatment with SYQP (25, 50, and 100 μg/ml) induced significant downregulation of iNOS and COX-2 mRNA and protein levels (Figures 3F–J). The DCFH-DA assay was used to quantify ROS levels. As depicted in Figure 5K, pre-treatment with DEX or SYQP led to marked reduction of reactive oxidative stress in cells exposed to LPS.

### SYQP Ameliorated LPS-Induced Pathological Consequences of Lung Tissues and Downregulated Inflammation Responsive Cells in BALF and Blood in LPS-Induced Acute Respiratory Distress Syndrome Mice

**FIGURE 4:**
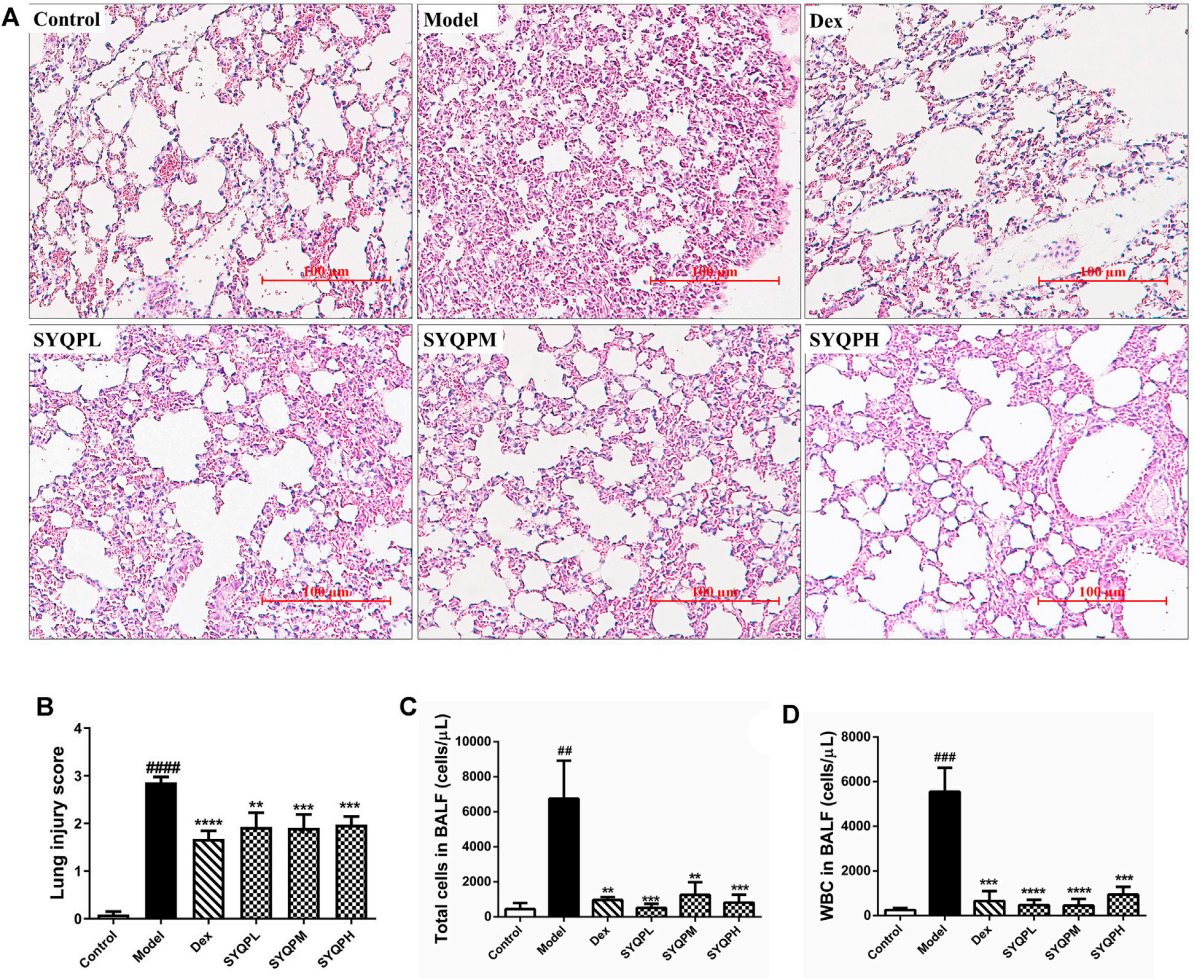
Effect of SYQP on histopathological and bronchoalveolar lavage (BAL) cytological changes of LPS-induced mice. Balb/c mice were divided into six groups: control group, LPS group (20 mg/kg), DEX (5 mg/kg) group, SYQPL (50 mg/kg) group, SYQPM (75 mg/kg) group, SYQPH (150 mg/kg) group. After intragastric administration of DEX or SYQP for 14 days in the administration group, LPS was injected intraperitoneally, and then mice were sacrificed after 12 h of LPS treatment. **(A)** Lung tissue sections were made and stained with Hematoxylin and Eosin. **(B)** Lung inflammation score in LPS-induced ARDS mice. **(C,D)** BALF was collected with 1 ml ice-cold PBS three times. Then BALF were combined and centrifuged for cell sorting and counting. The bars represent the mean ± SD (*n* = 6); ###*p* < 0.001 and ##*p* < 0.01 versus the control group; ****p < 0.0001, ***p < 0.001 and **p < 0.01 versus the model group.

**FIGURE 5:**
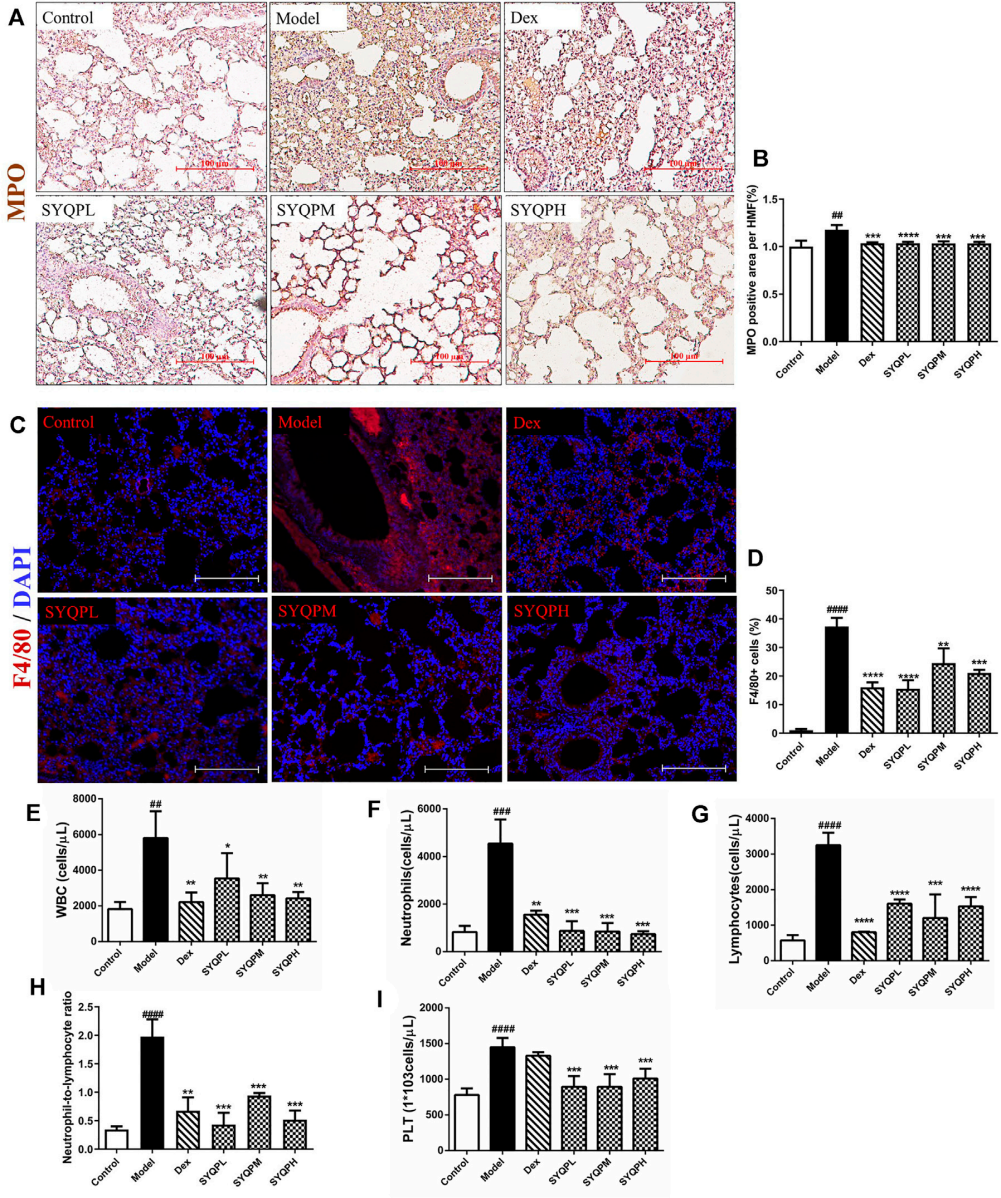
Effect of SYQP on MPO and F4/80 expression levels in lung tissues and hematological changes of LPS-induced mice. Animal experiment grouping and treatment were as described above, after the mice were sacrificed, the lung tissues of the mice were taken to make paraffin sections. **(A,B)** immunohistochemistry was used to label the expression levels of MPO. **(C,D)** immunofluorescence assay was used to label the macrophages. Red fluorescence represents F4/80-labeled macrophages and blue fluorescence represents DAPI-labeled nucleus. **(E–I)** The blood was collected in EDTA-K_2_ contained centrifugal tubes and subjected to automatic blood cell analyzer for detailed hematological analysis: **(E)** WBC, **(F)** neutrophils cells, **(G)** lymphocytes cells, **(H)** neutrophil-to-lymphocyte ratio and **(I)** platelets in LPS-induced ARDS mice. The bars represent the mean ± SD (*n* = 6); ####*p* < 0.0001, ###*p* < 0.001 and ##*p* < 0.01 versus the control group; ****p < 0.0001, ***p < 0.001, **p < 0.01 and *p < 0.05 versus the model group.

To verify the anti-inflammatory effects of SYQP in ARDS mice, we investigated the effect of SYQP on pulmonary morphological damage using HE staining. The results showed that normal pulmonary histology was seen in the control group. In contrast, lung tissues in LPS administration model group were significantly damaged, manifesting as thickening of the alveolar wall, interstitial edema, hemorrhage and infiltration of inflammatory cells, and the destruction was improved by DEX and SYQP treatment (Figures 4A,B). Next, we examined the BALF, and found that the total cells and white blood cells (WBC) in LPS-treatment group were elevated significantly. Whereas, SYQP and DEX treatment significantly inhibited the elevation of these cell counts in BALF (Figures 4C,D). As shown in Figures 5A,B, compared with model group, DEX and SYQP significantly reduced the expression level of myeloperoxidase (MPO), which is a key inflammatory enzyme (Shuang Chen et al., 2020) in the lung tissues of ARDS mice. In addition, the F4/80 immunofluorescence results indicated that pre-administration with DEX or SYQP could inhibited the macrophages in lung tissues of LPS-indued mice (Figures 5C,D). Moreover, whole blood analysis results showed that SYQP caused the reduction of the WBC, neutrophils cells, lymphocytes cells, the neutrophil-to-lymphocyte ratio (NLR) and platelets in LPS-induced ARDS mice compared with model group (Figures 5E–I). According to the BALF and whole blood quantitative analysis data, the anti-inflammatory function of SYQP was equivalent to or even better than DEX.

### SYQP Repressed the Production of GSH, SOD, and MDA and Inflammatory Signaling Responsive Cytokines in LPS-Induced Acute Respiratory Distress Syndrome Mice

**FIGURE 6:**
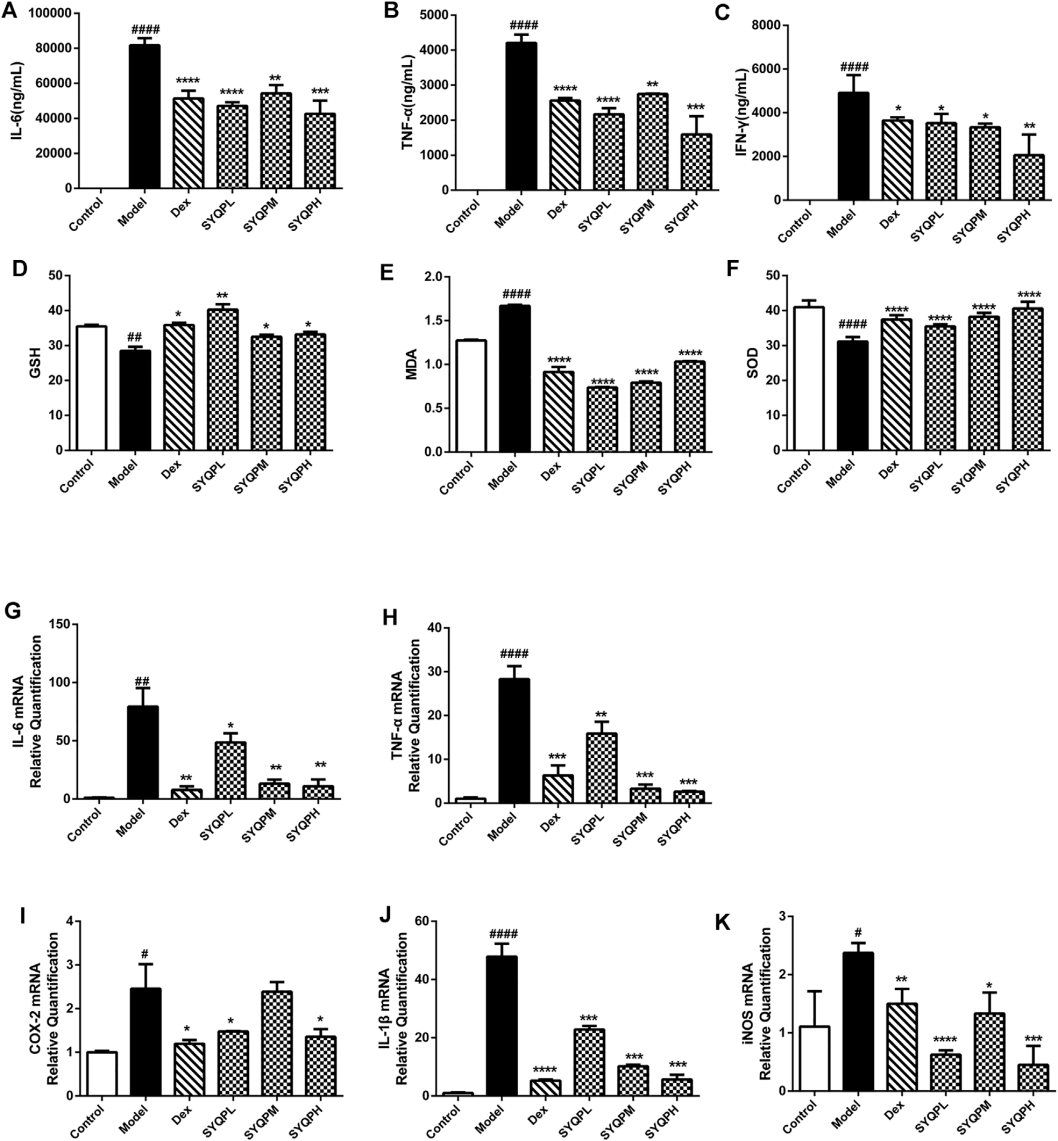
Effects of SYQP on the release and expression of pro-inflammatory cytokines, GSH, SOD, and MDA in ARDS mice. After treatment, plasma was collected by centrifugation and then stored at −80°C until pro-inflammatory cytokines, GSH, SOD and MDA measurement. **(A–C)** The cytokine contents in the plasma of ARDS mice were assayed using a Cytometric Bead Array Mouse Th1/Th2/Th17 Cytokine Kit. **(D–F)** The Effects of SYQP on GSH, SOD and MDA levels in plasma of ARDS mice were assayed by ELISA according to the manufacturer’s instruction. **(G–K)** Total RNA was isolated from lung tissues by RNA-Quick Purification Kit after the mice were sacrificed. After determining the concentration of the isolated RNA and reverse transcription, PCR was performed to determine the mRNA expression levels of pro-inflammatory cytokines, COX-2, and iNOS in lung tissues of ARDS mice. The bars represent the mean ± SD (*n* = 6); ####*p* < 0.0001, ###*p* < 0.001 and ##*p* < 0.01 versus the control group; ****p < 0.0001, ***p < 0.001, **p < 0.01 and *p < 0.05 versus the model group.

To investigate the effect of SYQP in LPS-induced ARDS mice, plasma and lung tissues samples were collected and GSH, SOD, MDA and cytokines were determined. As shown in Figures 6A–C, IL-6, TNF-α and IFN-γ levels were significantly increased in the plasma of the LPS group. Treatment of SYQP (50, 100, 150 mg/kg) to LPS-induced mice effectively reduced the cytokine secretion levels. In addition, SYQP significantly improved the levels of MDA, SOD and GSH in plasma of LPS-induced ARDS mice (Figures 6D–F). Additionally, we examined the mRNA levels of cytokines in lung tissues. The mRNA results showed that SYQP or DEX induced significant downregulation of IL-6, TNF-α, IL-1β, COX-2 and iNOS mRNA levels (Figures 6G–K).

### SYQP Inhibited TLR2/TLR4 Mediated Pulmonary Inflammation in LPS-Induced Acute Respiratory Distress Syndrome Mice

**FIGURE 7:**
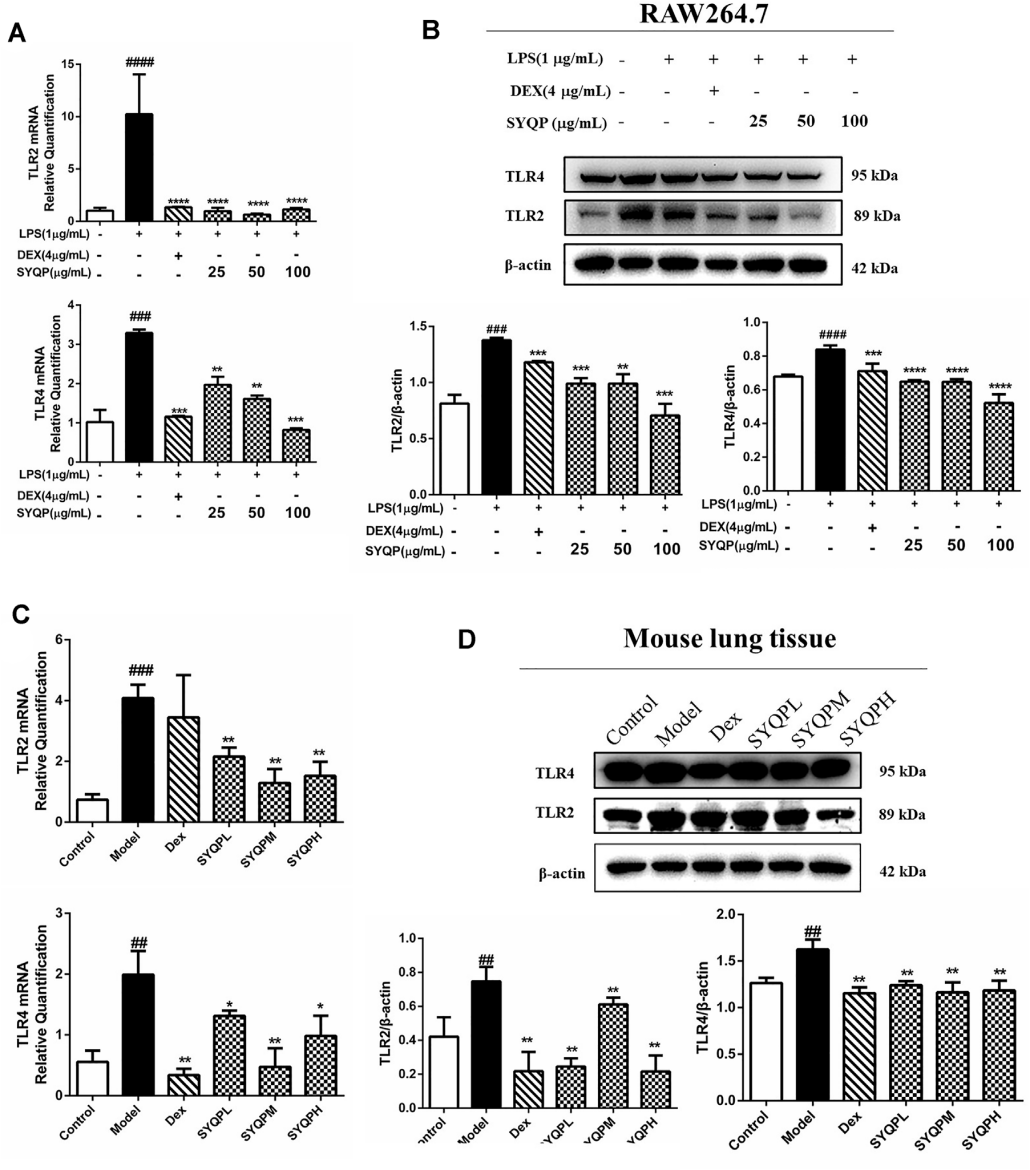
SYQP inhibited TLR2 and TLR4 gene and protein expression levels. **(A)** RAW264.7 cells were seeded in 6-well plates, pre-treated with a series of concentrations of SYQP or DEX (4 μg/ml) for 2 h, and followed by stimulation with or without LPS (1 μg/ml) for 18 h qRT PCR was used to detect the gene expression levels of TLR2 and TLR4. **(B)** Western blotting analysis was used to detect the protein expression levels of TLR2 and TLR4. **(C)** Total RNA was isolated from lung tissues by RNA-Quick Purification Kit after the mice were sacrificed. PCR was performed to determine the mRNA expression levels of TLR2 and TLR4 in lung tissues of ARDS mice. **(D)** The total proteins were extracted and analyzed by western blot to determine the protein expression levels of TLR2 and TLR4 in lung tissues of ARDS mice. The bars represent the mean ± SD (*n* = 3); ####*p* < 0.0001, ###*p* < 0.001 and ##*p* < 0.01 versus the control group; ****p < 0.0001, ***p < 0.001, **p < 0.01 and *p < 0.05 versus the model group.

To determine whether the anti-inflammatory effect of SYQP is related to the expression of TLR2/TLR4, we investigated the effects of SYQP on TLR2 and TLR4 in LPS-activated RAW264.7 cells and lung tissues of ARDS mice using Q-PCR and western blotting analysis. As illustrated in Figure 7, LPS strongly induced the expression levels of TLR4 and TLR2 *in vitro* and *in vivo*. and SYQP could significantly decreases TLR4 and TLR2 expression levels.

**FIGURE 8:**
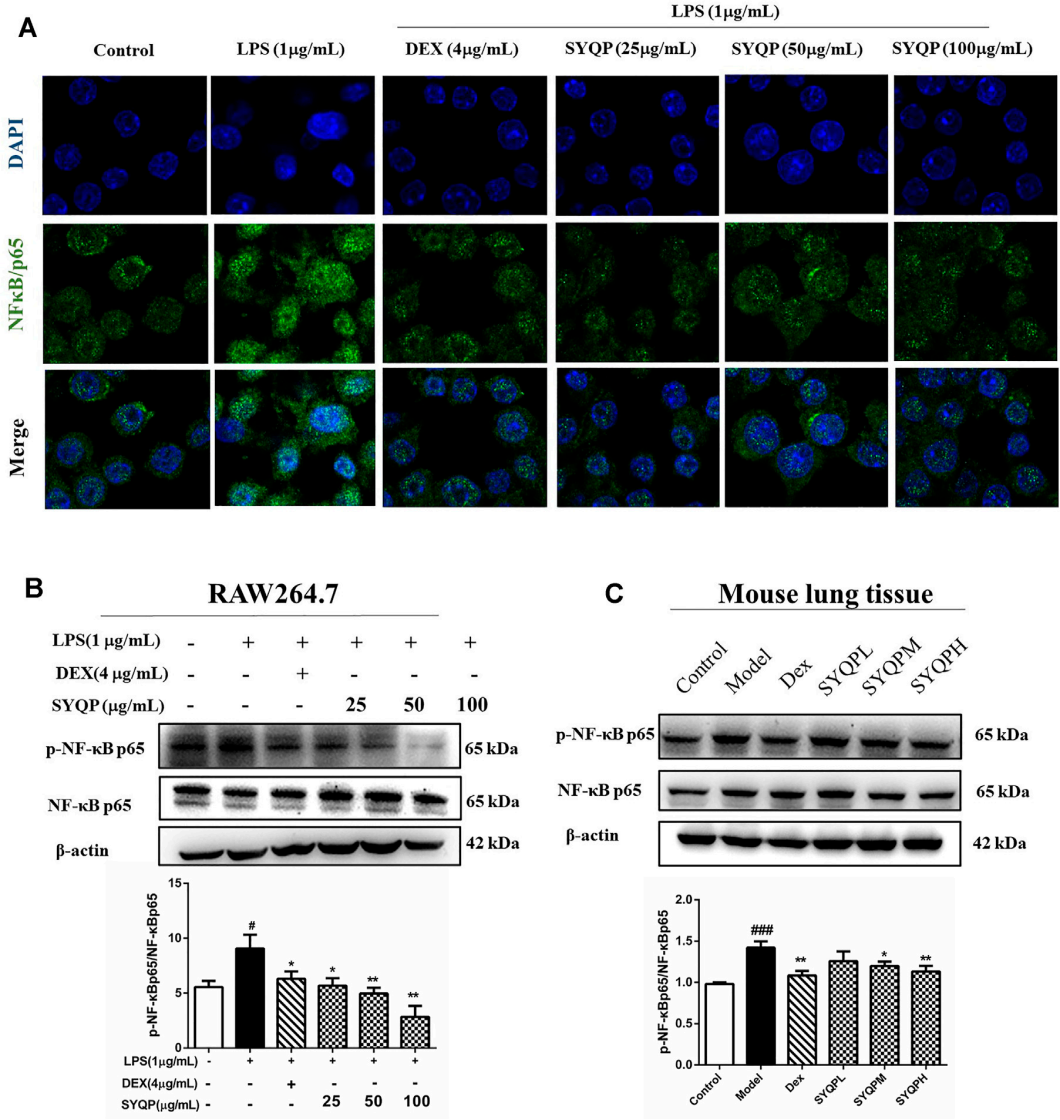
SYQP inhibited the nuclear translocation of NF-κB p65 and the phosphorylation of NF-κB p65. **(A)** RAW264.7 cells were seeded in Laser confocal Petri dishes, pre-treated with SYQP (25, 50, 100 μg/ml) or DEX (4 μg/ml) for 2 h, and followed by stimulation with or without LPS (1 μg/ml) for 18 h. After treatment, the nuclear translocation of NF-κB p65 of RAW264.7 cells was subjected to incubation with antibodies specific for NF-κB p65. Nuclei were co-stained for 10 min with 0.1 g/ml DAPI. Images were captured under laser scanning confocal microscope. **(B,C)** The total proteins were extracted and analyzed by western blot to determine the protein expression levels of the phosphorylation of NFκB p65 in LPS-induced RAW264.7 cells and lung tissues of ARDS mice. The bars represent the mean ± SD (*n* = 3); ####*p* < 0.0001, ###*p* < 0.001 and ##*p* < 0.01 versus the control group; ****p < 0.0001, ***p < 0.001, **p < 0.01 and *p < 0.05 versus the model group.

TLR2 and TLR4 signalling pathways culminate in activation of NF-κB and result in nuclear translocation of NF-κB p65 (Kawai and Akira, 2007). Hence, immunofluorescence analysis was performed to investigate whether SYQP could inhibit LPS-induced NF-κB p65 nuclear translocation in RAW264.7 cells. As shown in Figure 8A, SYQP prevented the NF-κB p65 transcription factor moving into the nucleus. We further observed the effects of SYQP on the phosphorylation of p65 NF-κB expression in LPS-induced RAW264.7 cells and lung tissues of ARDS mice using western blotting analysis. We found that SYQP inhibited the phosphorylation of p65 NF-κB expression levels *in vitro* and *in vivo* (Figures 8B,C). In addition, SYQP markedly inhibited the LPS-induced MyD88, phosphorylation of IKK-β and IκB-α expression in RAW264.7 cells (Supplementary Figure S2).

### SYQP Inhibited the Activation of TNF-α and IFN-γ Induced Inflammatory Death

**FIGURE 9:**
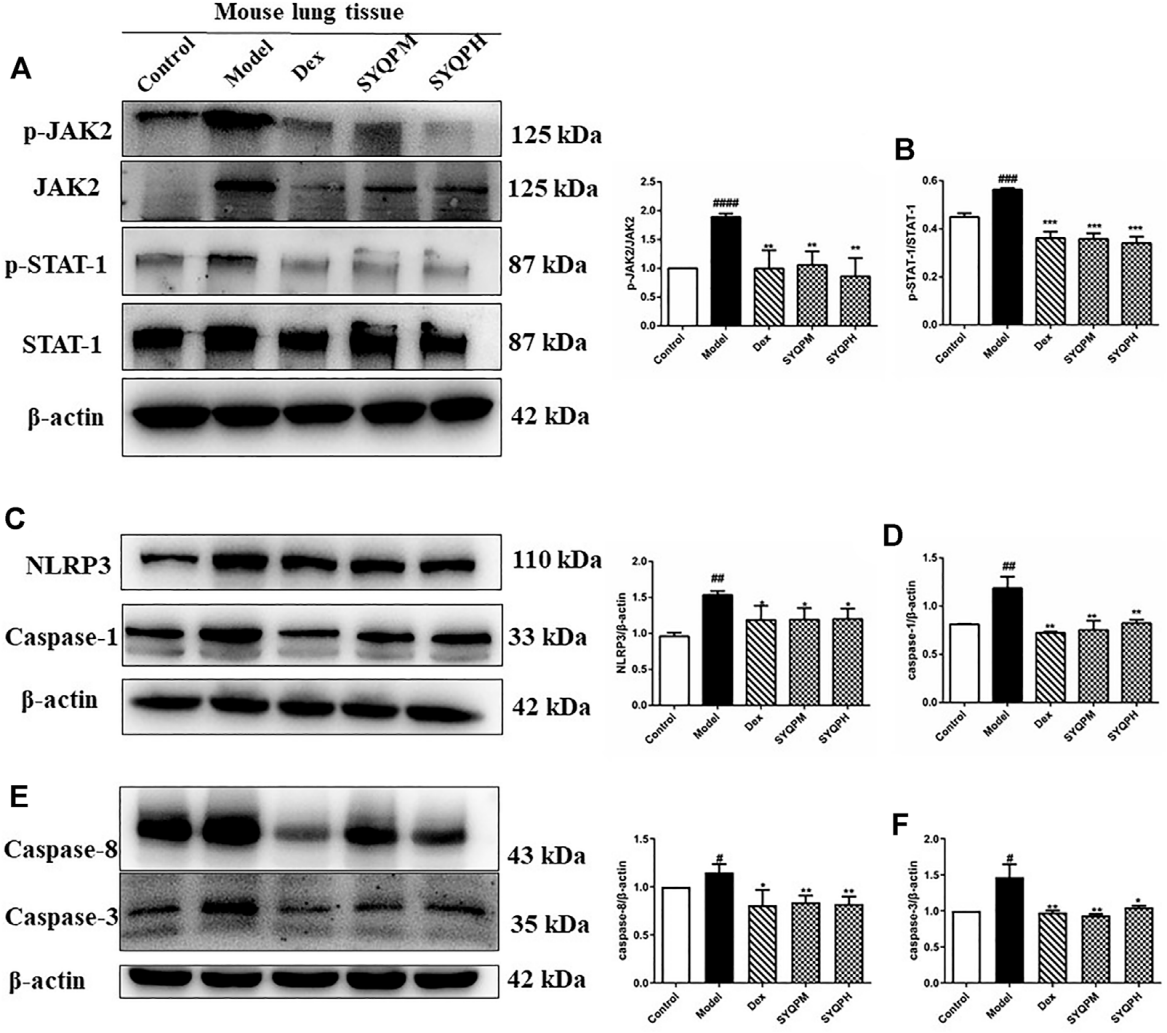
SYQP inhibited the activation of TNF-α and IFN-γ induced inflammatory death. The total proteins were extracted and analyzed by western blot to determine the protein expression levels in lung tissues of ARDS mice. **(A,B)** The protein expression levels of the phosphorylation of JAK2 and STAT-1. **(C,D)** The protein expression levels of NLRP3 and Caspase-1. **(E,F)** The protein expression levels of Caspase-8 and Caspase-3. The bars represent the mean ±SD (n=3); ####*p* < 0.0001, ###*p* < 0.001 and ##*p* < 0.01 versus the control group; ****p < 0.0001, ***p < 0.001, **p < 0.01 and *p < 0.05 versus the model group.

According to the results of *in vitro* and *in vivo* experiments, SYQP could effectively inhibit the expression levels of NO and iNOS in LPS-induced RAW264.7 cells, moreover, the secretion levels of TNF-α and IFN-γ in plasma of ARDS mice were significantly reduced by SYQP. Study results have shown that synergistic increase of TNF-α and IFN-γ could induce cell death through activating JAK/STAT1 axis, inducing nitric oxide production and driving caspase-8 -mediated PANoptosis (Karki et al., 2021). Therefore, we speculated that SYQP was effective in the protection of tissue damage in ARDS mice, and western blotting assay was used to confirm this viewpoint. As shown in Figures 9 A,B, the phosphorylation of JAK2 and STAT1 were significantly inhibited by SYQP, which were related to IFN-γ signaling pathway. What’s more, SYQP reduced the overexpression of NLRP3, caspase-1, caspase-3 and caspase-8 in lung tissues of ARDS mice (Figures 9C–F). These results revealed that SYQP could protect ARDS mice by reducing cell pyroptosis and even apoptosis.

## Dissussion

In this experiment, we mainly confirmed that SYQP induced bidirectional immunity *in vitro* and ameliorated LPS-induced ARDS in mice. Increasing evidence showed that most polysaccharides cannot directly enter cells due to their large molecular size (Lull et al., 2005; Xie et al., 2016), and the first step for polysaccharides to exert their functions is the recognition by pattern recognition receptors (PRRs) of cells (Wang et al., 2012; Barreto-Bergter and Figueiredo, 2014). The Toll-like receptor family is one of the most important families of PRRs (Wada and Makino, 2016) and plays critical roles in initiating innate inflammatory responses and promoting adaptive immune responses through different signaling pathways (Iwasaki and Medzhitov, 2010; Hoppstädter et al., 2012). In this study, experimental results of RAW264.7 cells indicated that SYQP stimulated the mRNA levels of TLR1, TLR2, and TLR6, but not TLR4. The observation was in keeping with reported literature that TLR2 gene expression was modified when cells were stimulated, while TLR4 levels were found to be unaffected (Paul-Clark et al., 2006). TLR2 must coactivates TLR1 or TLR6, forming heterodimers to evoke effects, and previous studies have demonstrated that polysaccharides improve the immune activity of macrophages due to their ability to bind to some PRRs, such as TLR2 and TLR4. The polysaccharides from Dendrobium huoshanense and Acanthopanax giraldii Harms were reported to have TLR4-mediated immunomodulatory activity (Xie et al., 2016; Li et al., 2019). TLR2 was the PRR of polysaccharides extracted from mushroom and Korean red ginseng (Lu et al., 2011; Byeon et al., 2012). Although our previous study has confirmed that SYQP stimulated TLR4 and showed immune adjuvant activity through stimulating TLR4 signaling pathway, according to literature study of polysaccharide and current experimental results, we proposed a new viewpoint that in addition to activating TLR4, SYQP could also activate TLR2/TLR1 or TLR2/TLR6, that is, SYQP could be used as ligands for different TLR receptors.

Studies have shown that both TLR2 and TLR4 ligands could activate NF-κB, and mitogen activated protein (MAP) kinases, while differentially releasing cytokines, such as TNF-α, IL-6, and IL-1β (Jones et al., 2001). TLR-neutralizing antibodies or TLR inhibitors could inhibit its related signaling activation and transduction. (Mistry et al., 2015; Garibotto et al., 2017). In order to study the effect of TLR2 and TLR4 on SYQP-activated immune cells, we measured the gene expression and secretion levels of pro-inflammatory cytokines in TLR2/TLR4 antagonist-treated RAW264.7 cells and MPMs after SYQP stimulation. The results showed that SYQP increased the expression levels of IL-6, TNF-α and IL-1β, however, individual pretreatment with C29 or TAK-242 decreased the stimulation of IL-6 and TNF-α production caused by SYQP, but cytokine secretion remained at a higher level compared with the control group. Combined application of C29 and TAK-242 almost completely abolished the stimulation of IL-6 and TNF-α production caused by SYQP. These results indicated that SYQP could induce macrophage immune response dependently of TLR2 and TLR4 activation. Our findings agreed with previous reports that polysaccharides obtained from the roots of Actinidia eriantha can activate the TLR2 and TLR4 pathways and promote the secretion of cytokines (Du et al., 2018). These above results demonstrated that TLR2 and TLR4 were correlated with the activation of macrophages treated by SYQP, which further clarified the mechanism of SYQP as macromolecular polysaccharides that exerted immunomodulatory effects.

It is well known that when macrophages were excessively activated by the severe infection, such as bacteria and viruses, the pro-inflammatory mediators cascade would break out. ARDS is a life-threatening disease associated with the migration of large numbers of inflammatory cells to the lungs, leading to the release of inflammatory mediators (Wilson and Saukkonen, 2004). Corticosteroids have potent anti-inflammatory effects and are considered as the first choice in the treatment of ARDS, however, their side effects largely limit the application. In these cases, the pharmacodynamics and mechanisms of TCM have attracted widespread research attention, and anti-inflammatory activities of polysaccharides derived from plants have been proved by more and more studies (Wang et al., 2014; Xie et al., 2018). It is notable that we conformed the antipyretic effect of SYQP in a previous study, which preliminarily verified the significant anti-inflammatory activity of SYQP (Bingqi Zhu et al., 2020). Hence, the effect of SYQP on ARDS mice and its possible mechanism turned out to be a meaningful topic and is worthy of investigation.

The activation of lung epithelial cells and lung macrophages could induce excessive ROS production, which may cause inflammation and pathobiological damage (Martin et al., 1997). Consistent with literature, we found that LPS promoted the overproduction of ROS in macrophages, whereas SYQP treatment significantly reduced the ROS levels. LPS or a large amount of cytokine stimulation could induce macrophages or lung tissue cells to promote the production of NO, the initiation of COX-2, the induction of iNOS (Hanafy et al., 2001; Korhonen et al., 2005; Knaapen et al., 2006). Our results demonstrated that SYQP sequestered the NO, iNOS and COX-2 gene and protein expression levels in LPS-induced macrophages. MPO is a kind of heme-containing peroxidase, which is highly expressed in a variety of inflammatory cells (Shuang Chen et al., 2020). We detected the expression of MPO in ARDS mice by immunohistochemistry, and found that SYQP significantly reduced the expression of MPO in lung tissues. In addition, SYQP improved the MDA, SOD and GSH levels in ARDS mouse plasma. Previous study demonstrated that polysaccharides extracted from *Tetrastigma hemsleyanum* tubers have similar anti-inflammatory effects (Chu et al., 2019). Combined with the previous immunostimulatory activity of SYQP, we preliminarily proved the dual immunomodulatory activity of SYQP.

The main pathophysiologic mechanisms of ARDS are uncontrolled inflammation in the lungs or the whole body. In this process, macrophages, neutrophils cells, lymphocytes cells and platelets accumulate in the lungs, activate cytokines, and disrupt the endothelial barrier (Janz and Ware, 2013; He et al., 2021). Previous study suggested that LPS could increase the number of inflammatory cells and induce the accumulation of serous fluids in the lungs (Card et al., 2006). Also, LPS induced hematological changes in ARDS mice (Whitehead et al., 2017). In the present study, we demonstrated that SYQP alleviated LPS-induced pulmonary morphological damage in mice, inhibited the number of macrophages in lung tissues, ameliorated the inflammatory cells in BALF, improved hematological status. Excessive production of cytokines leads to pathological damage and septic shock. We observed the increased expression of inflammatory cytokines such as TNF-α, IL-6, IL-1β and IFN-γ in LPS-induced macrophages and ARDS mice, their levels were significantly attenuated by SYQP treatment.

Pathogen-associated molecular pattern molecules (PAMPs) were recognized by diverse receptors on the cells of the innate immune system, including TLR and NLR to produce cytokines. Studies have shown that the excessive production of cytokines must rely on the co-activation of multiple coordinated innate system, and Root-Bernstein *et al.* proposed that synergistic interactions among TLR and NLR were involved in ARDS and sepsis (Root-Bernstein, 2021), for example, the activation of TLR2 and TLR4 could induce priming of the NLRP3 inflammasome, and NLRP3 activation could lead to pyroptosis (Coll et al., 2015; Kopitar-Jerala, 2015). In this study, SYQP significantly suppressed the excessive cytokine levels in ARDS mice, so we speculated that SYQP play immune protection through multiple signaling pathways. Härter *et al* reported that TLR2 and TLR4 were the main receptors upregulated during sepsis (Härter et al., 2004). In our study, we demonstrated that TLR2 and TLR4 were correlated with the activation of macrophages treated by SYQP. In addition, SYQP could reduce TNF-α levels in LPS-induced RAW264.7 cells and ARDS mice. TNF-α was one of the 20 members of the tumor necrosis factor superfamily (TNFSF), which was mainly released by stimulating TLRs, such as TLR2 and TLR4, and activating NF-κB (Paul-Clark et al., 2006; Liu et al., 2017). Hence, we firstly measured the mRNA and protein expression levels of TLR2 and TLR4. We found that LPS induced TLR2 and TLR4, whereas SYQP could inhibit the mRNA and protein expression levels of TLR2 and TLR4 in LPS-induced RAW264.7 cells and lung tissues of LPS-induced ARDS mice. The activation or TLRs could activate NF-κB signaling pathway, and our data suggested that SYQP decreased the phosphorylation of NF-κB, IKKβ and IκBα and prevented the NFκB p65 transcription factor moving into the nucleus. At present, many plant polysaccharides have been proven to have bidirectional immunomodulatory activity. Previous studies showed that Lentinan (Kupfahl et al., 2006; Nishitani et al., 2013), yupingfeng polysaccharide (Sun et al., 2017) and acidic extracellular polysaccharide produced by *Lactobacillus* (Wang et al., 2020) could not only promote the expression of cytokines in normal immune cells, but also inhibit the overexpression of cytokines in inflammatory immune cells. In this study, SYQP can promote the expression of IL-6 and TNF-α and IL-1β in macrophages, and reduced cytokine levels, including, IL-6, TNF-α and IFN-γ in LPS-induced mice and LPS-induced RAW264.7 cells, indicating bidirectional regulation of cytokine synthesis. It has been reported that several PRRs, especially TLR2 and TLR4 were the main receptors of polysaccharides in the regulation of macrophage activation and played bidirectional immunomodulatory roles (Zhang et al., 2013; Zhang et al., 2018; Yun Chen et al., 2020). The present study revealed that SYQP stimulate TLR2 and TLR4 expression in RAW264.7 cells. Moreover, in the LPS-induced RAW264.7 cells and ARDS mice, upregulation of TLR2 and TLR4 was significantly reversed by SYQP treatment. These results indicated that SYQP regulate the expression of TLR2 and TLR4 through dual immunomodulatory effects. What’s more, there were numerous studies that implicated the NLRP3 inflammasome in mediating inflammation and death during lung injury and ARDS (Freeman and Swartz, 2020; Morris et al., 2021). Therefore, we measured the protein expression levels of NLRP3/caspase family. It was reported that LPS could regulate NLRP3/ASC/caspase-1 inflammasome complex to activate lung macrophage pyroptosis in LPS-induced ALI model (Wu et al., 2015). In this study, the protein expression of NLRP3 and caspase-1 increased significantly after LPS treatment, which is consistent with previous studies. While SYQP treatment could decrease the expression levels of NLRP3 and caspase-1. In addition, the expression levels of caspase-3 and caspase-8 in ARDS mice lung tissues were inhibited by SYQP, which were related to pyrolysis and apoptosis pathways (Karki et al., 2021).

Moreover, we found a noteworthy phenomenon, whether it was intraperitoneal injection of SYQP in our previous study (Bingqi Zhu et al., 2020), or the oral absorption of SYQP for the treatment of ARDS in this study, consistent results were drawn that SYQP could significantly reduce IFN-γ levels. IFN-γ was the sole type ǁ IFN that plays physiologically important roles in promoting innate and adaptive immune responses (Ikeda et al., 2002). It was known that the autophosphorylation of JAK2 phosphorylated JAK1 to activate the transcription factor STAT1, which was located in the nucleus to induce the transcription of IFN-γ-responsive genes. Study have demonstrated that during the inflammatory process of ARDS, the synergism of TNF-α and IFN-γ could trigger inflammatory cell death, including pyroptosis and apoptosis, which involved multiple signal transduction pathways such as NLRP3/caspase, and JAK/STAT signaling pathways (Karki et al., 2021). We examined the survival rate of the LPS-induced ARDS mice after treating with DEX and SYQP. The mice in the control group all survived when the experiment ended at 72 h, while the survival rate of the mice in LPS group was only12.5%. The survival rate of mice pretreated with DEX and different concentrations of SYQP were increased (Data not shown). Therefore, SYQP effectively inhibited related signaling pathway may be an important reason for its effective treatment of ARDS. Western blot results suggested that the phosphorylation of JAK2 and STAT1 were significantly inhibited by SYQP, which were related to IFN-γ signaling pathway. Similar results were obtained by Han et al. (2020), who reported that polysaccharides isolated from young barley leaves were effective in improving the immunological manifestations through JAK/STAT1 signaling pathway. In the follow-up experiments, we would further explore the protective effect of SYQP on cell death, tissue damage, and mortality *in vitro* and *in vivo*, in order to fully understand the application of SYQP in ARDS.

In summary, the present study showed that the bidirectional immunity of SYQP could activate or inhibit the TLR2 and TLR4 signalling pathways at the receptor level and revealed TLR2/TLR4 modulatory characteristics. On the one hand, SYQP stimulated the gene expression of TLR1, TLR2, TLR4 and TLR6, and promoted the secretion of IL-6 and TNF-α through TLR2/TLR4. On the other hand, SYQP could inhibit LPS-induced excessive inflammation in RAW264.7 cells and LPS-induced ARDS mice through TLR2/TLR4-NFκB, NLRP3/caspase and JAK/STAT signaling pathways. This study provided a scientific basis for developing SYQP as a clinical immunomodulator for functional foods or medicines, and in-depth elucidation of the protective effect of SYQP on cell death, tissue damage, and mortality would be important subjects for further investigation.

## Data Availability

The original contributions presented in the study are included in the article/Supplementary Materials, further inquiries can be directed to the corresponding author.
