# Supplementary figures and images for: Polysaccharides From the Aerial Parts of Tetrastigma Hemsleyanum Diels et Gilg Induce Bidirectional Immunity and Ameliorate LPS-Induced Acute Respiratory Distress Syndrome in Mice
Source: Front Pharmacol. 2022 Mar 11;13:838873. doi: 10.3389/fphar.2022.838873 (PMC8965720; doi:10.3389/fphar.2022.838873)

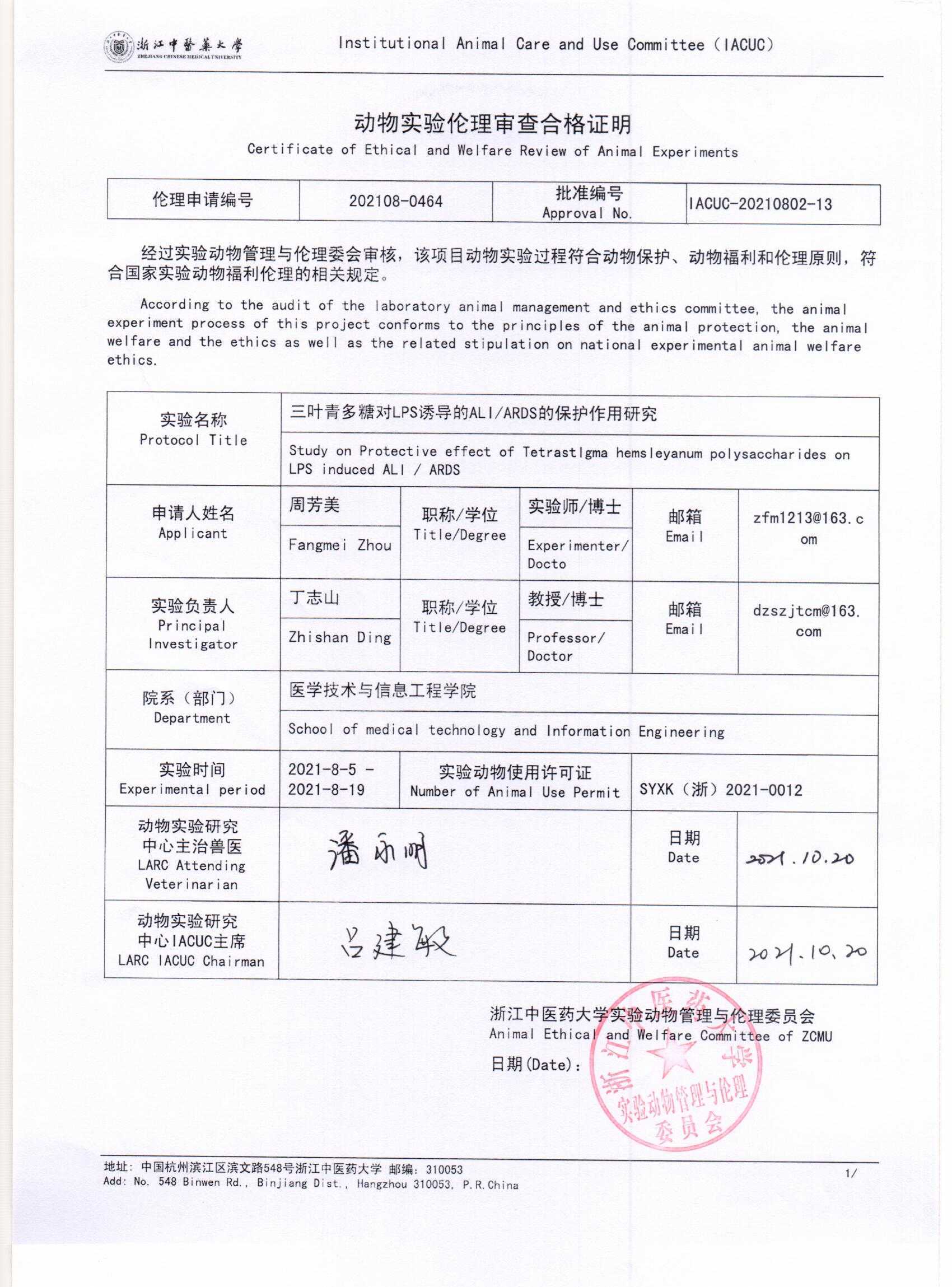

Supplement: Supplementary file 1 [file Image1.JPEG]
