# Supplementary material for: Polysaccharides From the Aerial Parts of Tetrastigma Hemsleyanum Diels et Gilg Induce Bidirectional Immunity and Ameliorate LPS-Induced Acute Respiratory Distress Syndrome in Mice
Source: Front Pharmacol. 2022 Mar 11;13:838873. doi: 10.3389/fphar.2022.838873 (PMC8965720; doi:10.3389/fphar.2022.838873)
Supplement: Supplementary file 2 [file DataSheet1.docx]

Supplementary Material

## Supplementary Figures
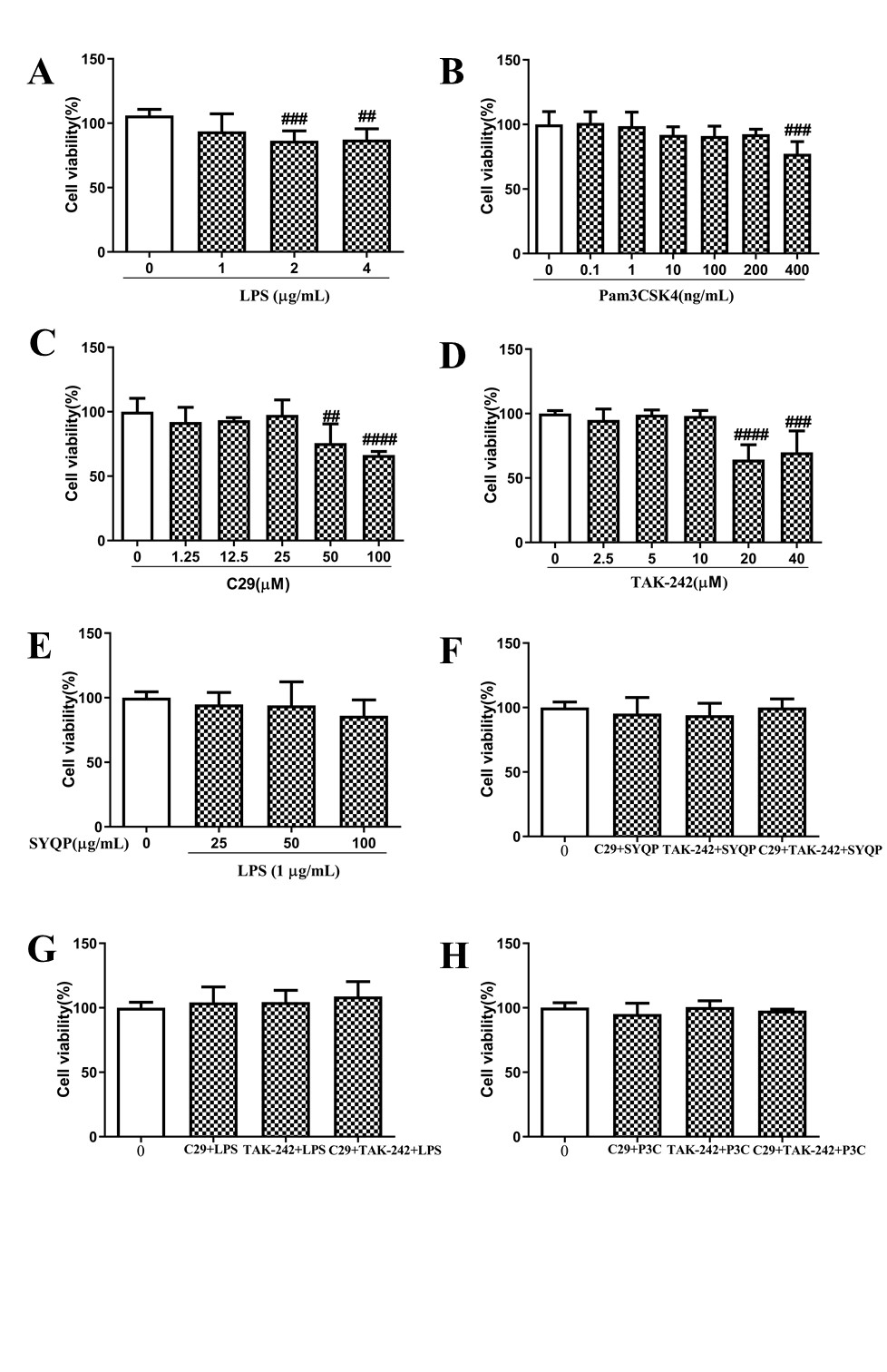


Supplementary Figure 1. Evaluation of cell viability on RAW264.7 cells. Data were presented as the mean ± SD (n=9); **####**p<0.0001, ###p<0.001 and ##p<0.01 versus the control group.


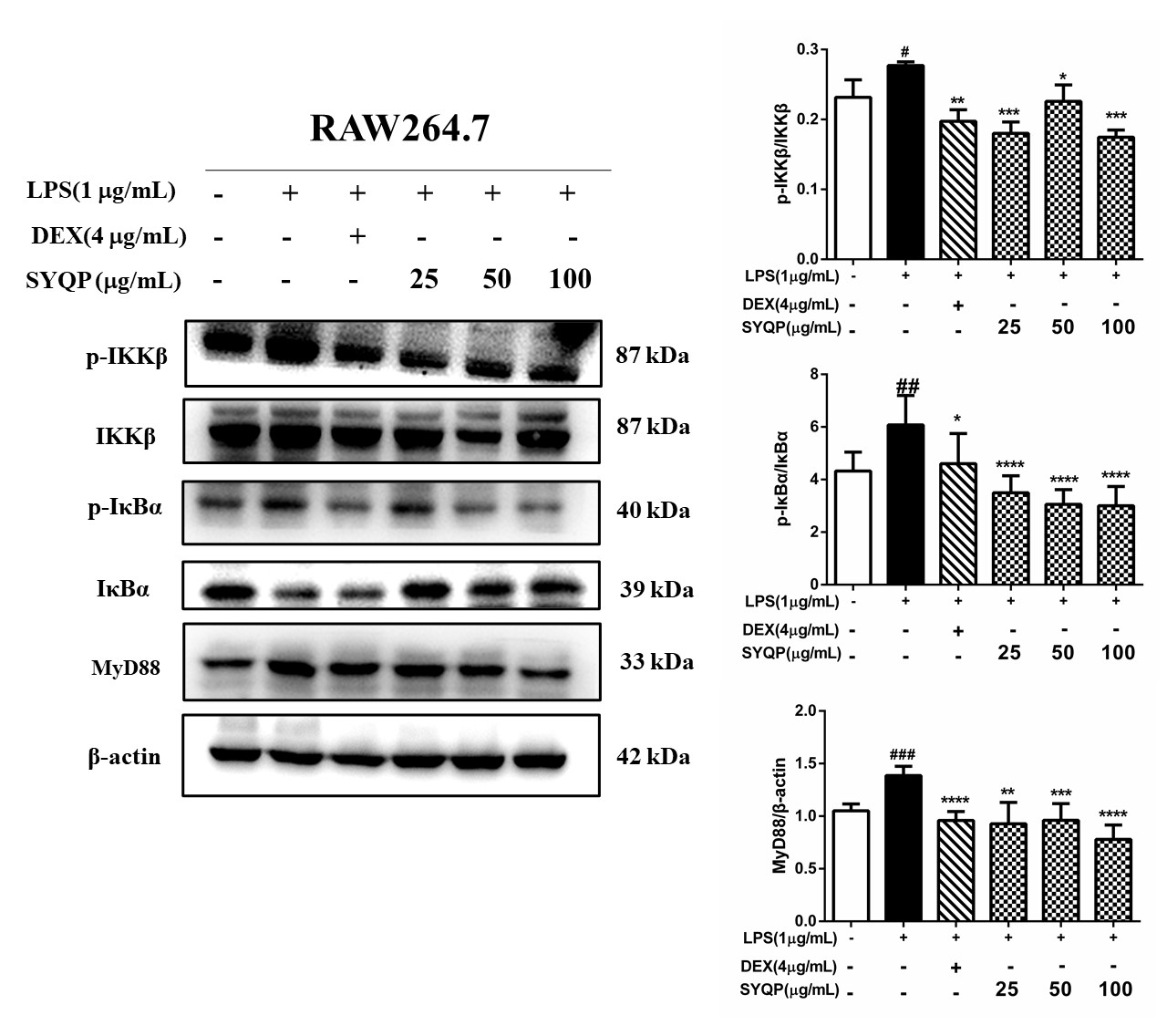


Supplementary Figure 2. SYQP inhibited the proteins levels of MyD88and the phosphorylation of IKKβ, IκBα. RAW264.7 cells were seeded in Laser confocal petri dishes, pre-treated with SYQP (25, 50, 100 μg/mL) or DEX (4 μg/mL) for 2 h, and followed by stimulation with or without LPS (1 µg/mL) for 18 h. After treatment, the total proteins were extracted and analyzed by western blot to determine the protein expression levels in LPS-induced RAW264.7 cells. The bars represent the mean ±SD (n=3); ####*p*<0.0001, ###*p*<0.001 and ##*p*<0.01 versus the control group; *****p*<0.0001, ****p*<0.001, ***p*<0.01 and **p*<0.05 versus the model group.
